# Algorithm-Based Modular Psychotherapy Alleviates Brain Inflammation in Generalized Anxiety Disorder

**DOI:** 10.3390/life14070887

**Published:** 2024-07-18

**Authors:** Szabolcs Kéri, Alexander Kancsev, Oguz Kelemen

**Affiliations:** 1 Sztárai Institute, University of Tokaj, 3944 Sárospatak, Hungary; 2 Department of Physiology, Albert Szent-Györgyi Medical School, University of Szeged, 6720 Szeged, Hungary; 3 Department of Psychiatry and Psychotherapy, András Jósa Hospital, 4400 Nyíregyháza, Hungary; alexander.kancsev@phd.semmelweis.hu; 4 Department of Behavioral Sciences, Albert Szent-Györgyi Medical School, University of Szeged, 6720 Szeged, Hungary; kelemen.oguz@med.u-szeged.hu; 5 Department of Psychiatry and Psychotherapy, Bács-Kiskun County Hospital, 6000 Kecskemét, Hungary

**Keywords:** generalized anxiety disorder, cognitive–behavioral therapy, modular psychotherapy, neuroinflammation, amygdala

## Abstract

Generalized anxiety disorder (GAD) is marked by prolonged and excessive worry, physical signs of anxiety, and associated neuroinflammation. Traditional treatments, like pharmacotherapy and cognitive–behavioral therapy (CBT), often leave residual symptoms and have high relapse rates. This study aimed to explore the efficacy of algorithm-based modular psychotherapy (MoBa), a combination of CBT and mindfulness meditation as validated by the research domain criteria (RDoC), in reducing anxiety and neuroinflammation in GAD. A longitudinal design was used, with 50 patients with GAD undergoing a 12-week MoBa treatment. The patients were investigated pre- and post-treatment using MRI to measure neuroinflammatory markers (DBSI-RF, diffusion-basis spectral imaging-based restricted fraction) in the hippocampus, amygdala, and neocortex. Clinical symptoms were assessed using the Hamilton Anxiety Rating Scale (HAM-A) and the Generalized Anxiety Disorder 7-item scale (GAD-7). Results indicated significant reductions in both anxiety symptoms and MRI RF values in the amygdala, suggesting decreased neuroinflammation. A reduction in anxiety was associated with the amelioration of neuroinflammation in the amygdala. These results suggest that MoBa is effective in alleviating both the psychological and neuroinflammatory aspects of GAD, offering a promising personalized treatment approach. Future research should focus on long-term effects and the mechanisms through which MoBa impacts neuroinflammation and anxiety.

## 1. Introduction

Generalized Anxiety Disorder (GAD) is characterized by chronic, excessive, and uncontrollable worry, accompanied by restlessness, physical and mental fatigue, poor concentration, irritability, muscle tension, and sleep disturbances [1]. GAD affects approximately 3.1% of the population annually in the United States, making it one of the most common anxiety disorders [2]. GAD impacts the patients’ daily functioning and quality of life, underscoring the urgent need for more effective treatments [3,4].

Traditional treatments for GAD include pharmacotherapy and various forms of psychotherapy. Antidepressants and benzodiazepines are commonly prescribed medications that have shown efficacy in reducing anxiety symptoms, but the side effects and the risk of addiction limit their application [5,6,7]. Cognitive–behavioral therapy (CBT) is the most frequently studied and utilized psychotherapeutic approach, demonstrating significant benefits in numerous clinical trials [8,9,10]. However, despite these available treatments, a substantial proportion of patients with GAD continue to experience residual symptoms, and relapse rates remain high [4].

The mechanism of residual symptoms and clinical relapse tendencies is multifaceted. Recent research has increasingly focused on the role of neuroinflammation in the pathophysiology of anxiety disorders, including GAD [11,12,13,14,15]. During neuroinflammation linked to negative affective states, the brain’s immune response is dysregulated, characterized by the release of pro-inflammatory cytokines and other mediators that disrupt neuronal information processing and metabolism [16,17,18,19]. Chronic and subtle inflammation in the brain has recently been implicated in various psychiatric conditions [20,21]. Accordingly, studies repeatedly reported elevated levels of C-reactive protein (CRP), interleukin-6 (IL-6), and tumor necrosis factor-alpha (TNF-α) in individuals with GAD compared to healthy controls [13,15,22]. CRP and IL-6 levels are associated with specific anxiety symptoms, such as irritability and worrying [12].

Inflammation affects brain regions involved in affect regulation and fear responses, such as the amygdala, hippocampus, insula, and anterior cingulate cortex, potentially contributing to anxiety [17,20,23]. The hippocampus and amygdala are important in regulating emotion, memory formation, and stress responses, which are relevant to the symptoms of GAD [24,25,26]. The hippocampus is essential for engram formation and contextualizing emotional responses, while the amygdala plays a crucial role in threat detection and generating fear and anxiety [24,27,28]. The amygdala is consistently found to be hyperactive in individuals with GAD. This hyperactivity is linked to heightened emotional responses and persistent anxiety [29]. The hippocampus often shows structural and functional abnormalities in patients with GAD. These abnormalities can contribute to the cognitive aspects of anxiety and to a lessened ability to regulate stress responses effectively [30]. There is evidence of disrupted functional connectivity between the amygdala and hippocampus, which affects how these regions communicate and coordinate their roles in stress and anxiety responses [31]. The anterior cingulate cortex and ventral hippocampus projections to the basolateral amygdala regulate generalized fear responses, a common symptom in GAD. Dysregulation in these projections can lead to overgeneralizing fear in non-threatening contexts [32]. Patients with anxiety disorders often exhibit structural abnormalities in the extended limbic structures. For example, patients with generalized social phobia displayed reduced amygdala and hippocampus volume, and the latter was associated with the severity of anxiety [33]. 

Studies have shown that neuroinflammation can affect the structure and function of these brain regions, potentially exacerbating anxiety symptoms [34]. For example, inflammation in the hippocampus has been associated with impaired neurogenesis and synaptic plasticity, which can contribute to cognitive deficits and mood disturbances [35]. Similarly, inflammation in the amygdala can enhance its reactivity to stressors, leading to heightened anxiety and emotional dysregulation [23,36]. Thus, neuroinflammation, specifically within the amygdala, can lead to increased anxiety-like behavior. This is often mediated through pro-inflammatory cytokines, which enhance excitatory synaptic transmission while inhibiting inhibitory synaptic transmission, disrupting the excitatory-inhibitory balance [37,38]. Increased inflammation, marked by elevated levels of CRP and inflammatory cytokines, is associated with decreased functional connectivity between the amygdala and the ventromedial prefrontal cortex. Weakened connectivity correlates with heightened anxiety symptoms [39]. Moreover, chronic stress induces a pro-inflammatory state that increases microglial activation and neuronal activity in the amygdala, which is associated with anxiety [40]. The CXCL12/CXCR4 pathway in the amygdala is implicated in the development of anxiety induced by systemic inflammation. Blocking this pathway can reduce anxiety-like behaviors [41]. Recently, it has been shown that inflammatory responses in the hippocampus, triggered by factors, such as bacterial lipopolysaccharide, increase inflammatory cytokine expression (e.g., IL-1β, TNF-α). This inflammation is associated with anxiety-like behaviors and altered gene expression, indicating a direct link between hippocampal inflammation and anxiety [42].

An effective treatment of GAD that meets the needs of patients is an unresolved issue. Algorithm-based modular psychotherapy (MoBa) represents a novel therapeutic approach that integrates various evidence-based psychotherapeutic techniques into a modular framework tailored to the patient’s needs [43,44,45,46]. This method utilizes algorithms to guide the selection and sequencing of therapeutic modules, such as CBT [8], mindfulness and acceptance-based interventions [47], and emotion-focused therapy [48], to address the unique symptom profile and underlying mechanisms of each patient with GAD. MoBa’s flexibility and personalized nature make it a promising approach for improving treatment outcomes in GAD.

The potential of MoBa to reduce neuroinflammation and thereby alleviate anxiety symptoms has never been investigated. By targeting the underlying inflammatory processes in the brain, MoBa may offer a more holistic approach to treating GAD. This hypothesis is supported by evidence showing that various psychotherapeutic techniques can modulate inflammatory responses [49,50,51,52,53]. For example, mindfulness-based interventions reduce the levels of pro-inflammatory cytokines and improve immune function [50]. Similarly, CBT has been associated with reductions in inflammation, as indicated by lower concentrations of CRP and other peripheral inflammatory markers in mood and anxiety disorders [51]. Generally, psychosocial interventions may improve immune functions and reduce inflammation, although the results are still tentative and inconclusive [54,55,56,57].

The present study aims to investigate the impact of MoBa on central neuroinflammatory markers in patients with GAD, focusing specifically on the hippocampus and amygdala. Novel magnetic resonance imaging (MRI) techniques can provide detailed structural and functional information about the hippocampus and amygdala, as well as indirect measures of neuroinflammation. The MRI DBSI-RF (diffusion-basis spectral imaging-based restricted fraction) approach detects the altered diffusion properties of water around nerve fibers, indicating axonal injury, demyelination, and inflammation [58]. DBSI-RF results from human volunteers are in accordance with post-mortem cellular indicators in neuroinflammatory conditions (multiple sclerosis, obesity, and Alzheimer’s disease) [59,60,61]. Moreover, in individuals experiencing depressive symptoms, elevated CRP was associated with a higher MRI-restricted fraction (RF) [36,62].

The primary objective of this study is to determine whether MoBa can reduce inflammation in the hippocampus and amygdala, thereby alleviating symptoms of GAD. By combining neuroimaging data with clinical assessments of anxiety, this research seeks to elucidate the relationship between psychotherapy and neuroinflammatory processes. Understanding how MoBa influences brain inflammation could reveal new pathways for therapeutic intervention and contribute to developing more effective, personalized treatments for GAD.

Specifically, we put forward two main hypotheses: (1) patients with GAD show decreased MRI inflammatory markers in the hippocampus and amygdala after MoBa; (2) significant normalization of MRI neuroinflammatory markers is associated with more improvement in anxiety symptoms.

## 2. Materials and Methods

### 2.1. Study Design

This study implemented a longitudinal, within-subject design to assess the effects of MoBa in patients with GAD. Participants were evaluated at baseline (pre-treatment) and after a 12-week course of MoBa (post-treatment). The primary outcomes were changes in MRI neuroinflammatory markers (RF) in the hippocampus and amygdala, as well as clinical symptoms of anxiety (post-treatment minus pre-treatment). The study also included a group of control volunteers without mental disorders. The control participants were also evaluated twice to prove the consistency of MRI markers. 

### 2.2. Participants

The patients with GAD (*n* = 50) were recruited from outpatient psychiatric clinics and through online advertisements at the National Psychiatric Center in Budapest and the University of Szeged, Hungary. The inclusion criteria were as follows: fulfilling the Diagnostic and Statistical Manual of Mental Disorders (DSM-5) criteria of GAD; age between 18 and 65 years; no significant lifetime psychiatric comorbidities (e.g., major depressive disorder, bipolar disorder, psychotic disorders); never receiving any form of psychotherapy or psychotropic medications before the study. The exclusion criteria included neurological disorders or head injury (any medical conditions affecting the central nervous system), substance use within the past six months, pregnancy or breastfeeding, using anti-inflammatory drugs within the past six months, and any general contraindications to MRI. All 50 patients with GAD completed the study. 

We also enrolled 50 control participants without prior psychiatric history or current complaints who were matched to the patients with GAD for age, sex, education, and potential confounding variables affecting inflammation (nicotine, caffeine, and alcohol intake, contraception use, body mass index (BMI), chronic diseases, and working night shifts) [63] (Table 1). We used social media advertisements to recruit the control participants. During the selection process, we considered the above-mentioned characteristics of the GAD group to select the appropriate control volunteers. All participants, including patients with GAD and controls, were Caucasian and declared themselves cisgender (he/him and she/her).

In the enrollment phase, 12 patients with GAD were not entered into the study because of comorbidities, and 8 control individuals were excluded because of mental disorders or inflammatory diseases. 

### 2.3. Clinical Assessment

All volunteers who were enrolled in the present study, including the control participants, received the structured clinical interview for DSM-5, administered by a trained assessor blind to the study’s aim [64].The assessors received financial compensation for their work and were not informed about the study’s aims and design. They were also not informed who they examined (the potential GAD group or the non-clinical control group). This interview was used to establish the diagnosis of GAD and to exclude comorbidity. Anxiety symptoms were evaluated using the Hamilton Anxiety Rating Scale (HAM-A) [65] and the Generalized Anxiety Disorder 7-item scale (GAD-7) [66]. Participants also completed the Beck Depression Inventory-II (BDI-II) [67] to control for depressive symptoms. The scales were administered before and after MoBa. 

The HAM-A is based on a semi-structured interview to assess the intensity of anxiety symptoms across 14 items, each rated from 0 (not present) to 4 (severe) (total score: 0–56). The scale evaluates both psychic anxiety (mental and psychological distress) and somatic anxiety (physical symptoms). Scoring is categorized into severity ranges: 0–17 indicates mild anxiety, 18–24 indicates moderate anxiety, and 25–30 indicates severe anxiety [65]. We observed a high internal consistency (Cronbach’s alpha: 0.90) and test–retest reliability for HAM-A (r = 0.84). The Generalized Anxiety Disorder 7-item (GAD-7) scale is a self-report questionnaire used to screen for the severity of GAD. It includes seven items, each rated from 0 (not at all) to 3 (nearly every day) (total score: 0–21). The severity of anxiety is classified as minimal (0–4), mild (5–9), moderate (10–14), and severe (15–21) [66]. Similar to HAM-A, we observed a high internal consistency (Cronbach’s alpha: 0.92) and test–retest reliability for GAD-7 (r = 0.81). The BDI-II is also a self-report questionnaire evaluating the severity of depression. It comprises 21 items, each rated on a scale from 0 to 3, reflecting the intensity of symptoms experienced over the past two weeks. The total score ranges from 0 to 63 (minimal: 0–13, mild: 14–19, moderate: 20–28, and severe: 29–63) [67]. Finally, we observed excellent internal consistency (Cronbach’s alpha: 0.93) and test–retest reliability for BDI-II (r = 0.91).

### 2.4. Interventions

Participants underwent a 12-week course of MoBa during which they did not receive psychotropic medications. This protocol integrates various evidence-based therapeutic modules, including standard CBT for GAD and other modules targeting patients’ needs [68,69,70]. To select the additional modules for CBT, we implemented the algorithm defined by the US National Institute of Mental Health Research Domain Criteria (RDoC) (negative valence systems, positive valence systems, cognitive systems, systems for social processes, arousal/regulatory systems, and sensorimotor systems) using a standard template for the psychiatric review of systems based on interviews and clinical records [71]. The MoBa approach consists of three primary modules focused on deficits related to early childhood maltreatment: systems of negative valence, social processes, and arousal. These modules integrate elements from the cognitive behavioral analysis system of psychotherapy (CBASP), mentalization-based psychotherapy (MBT), and mindfulness-based cognitive therapy (MBCT). Patients are treated in 20 individual sessions during the 12 weeks (two sessions in the first eight weeks), where the selection and application of modules are tailored to their specific needs using a questionnaire-based treatment algorithm. The algorithm ensures that therapy is personalized by selecting the appropriate modules based on the patient’s specific psychopathological mechanisms and needs. In the negative valence system, assessed with the Rejection Sensitivity Questionnaire, the objective is to modify the social threat response system and reduce avoidance behavior using elements from the CBASP. In the social processes system, as measured with the Interpersonal Reactivity Index, the aim is to enhance the perception and understanding of self and other’s mental states and to facilitate social communication using mentalization elements. Finally, in the arousal system, evaluated with the Difficulties in Emotion Regulation Scale, the therapist reduces hyperarousal by fostering emotional awareness and regulation by applying mindfulness-based elements [43,46]. 

Potential and sustained threats (negative valence systems) and hyperarousal were typical RDoC features in the GAD sample. In addition to the standard CBT, we applied mindfulness meditation modules, including simple stretches, postures, and emotion awareness and regulation techniques [69,70]. 

The psychotherapists (psychiatrists or clinical psychologists) working with patients with GAD received formal training in CBT and mindfulness-based techniques. Randomly selected therapy sessions were video-recorded and reviewed by independent supervisors to ensure the fidelity to the treatment protocol. The psychotherapists and supervisors were blind to the study’s aim. 

### 2.5. Magnetic Resonance Imaging (MRI)

Diffusion-weighted imaging (DWI) and T1-weighted structural scans were acquired following the United Kingdom (UK) biobank protocol [36,72,73]. Images were processed with FreeSurfer v7.4.1 [74]. The scanning parameters were as follows: Philips Achieva 3T scanner, MPRAGE (magnetization-prepared rapid acquisition gradient echo), 3D sagittal acquisition, FOV (square field of view) = 5256 mm, 1 × 1 × 1 mm^3^, TI = 5900 ms, TE (shortest) = 3.16, flip angle: 9 degrees, no fat suppression, full k space, no averages, acquisition time: 6 min and 50 s, acceleration factor: 2. For the DWI data, we used a multi-shell approach (b1 = 1000 s/mm^2^, b2 = 2000 s/mm^2^, 2 × 2 × 2 mm^3^, 50 diffusion encoding directions for each shell). We used eddy currents and head motion corrections, outlier slice correction, and gradient distortion correction during DWI preprocessing [36,72]. Putative neuroinflammatory changes were quantified by restricted fraction (RF) from DWI data (diffusion-basis spectral imaging-based restricted fraction, DBSI-RF) [58,61]. We focused on the hippocampus and amygdala using FreeSurfer regions of interest (ROIs). We extracted DBSI-RF from these ROIs [36,75,76]. The left and right RF values were averaged because they showed high lateralized correlations (left–right correlations: rs > 0.8) [36]. The whole gray matter of the cortex served as a control condition [36,77]. 

### 2.6. Data Analysis

We used the STATISTICA 13.1 platform (StatSoft) for data analysis. In order to achieve a statistical power of 80%, a sample size of *n*  =  63 would have been needed in each group (GAD vs. controls) to detect statistically significant differences at the α = 0.05 level.

Following basic descriptive statistics and data quality checks (calculation of means, standard deviation, skewness, kurtosis, normal distribution (Kolmogorov–Smirnov test), and homogeneity of variance (Levene’s test)), repeated-measures analysis of variance (ANOVA) was performed on the MRI RF values. The between-subjects factor was the experimental group (GAD vs. controls), and the within-subjects factors were testing time (before and after treatment) and brain regions (amygdala, hippocampus, and neocortex). The ANOVA effect size values were determined (partial eta-squared). Tukey’s honestly significant difference (HSD) tests were used for post hoc comparisons. 

We also investigated the relationship between changes in MRI RF values during treatment and changes in clinical symptoms. For these purposes, we first calculated the difference scores in RF, HAM-A, GAD-7, and BDI-II data (values after the treatment minus values before the treatment). A partial correlation approach was used where each comparison was corrected for age, education, and BMI. The FDR (false discovery rate) approach was used to correct multiple comparisons (nine correlation coefficients between differential RF values in the amygdala, hippocampus, and neocortex, and differential scores of HAM-A, GAD-7, and BDI-II). The clinical and demographic variables were analyzed with two-tailed *t*-tests and chi-square tests (sex distribution and the ratio of smokers and non-smokers in the GAD and control groups). The effect of sex was investigated in two ways: sex as a covariate in the analyses and by directly comparing male and female participants for clinical scales and MRI measures. For the clinical measures, Cohen’s effect size values (*d*) were calculated. The pre-corrected level of statistical significance was set at alpha < 0.05.

## 3. Results

### 3.1. Clinical Symptoms

Table 1 depicts the HAM-A, GAD-7, and BDI-II scores before and after treatment. We observed significant improvements in general anxiety (HAM-A), GAD-related symptoms (GAD-7) and depressive symptoms (BDI-II). There was a significant reduction in HAM-A scores during MoBa with a large effect size (*t*(98) = 4.19, *p* < 0.01, *d* = 0.88), similar to GAD-7 (*t*(97) = 5.32, *p* < 0.001, *d* = 1.1), revealing that MoBa was effective in reducing anxiety levels in patients. In addition, we observed improvements in subclinical depressive symptoms, as indicated by the BDI-II scores (*t*(98) = 2.25, *p* < 0.05, *d* = 0.45) (Table 1). 

### 3.2. MRI Markers of Neuroinflammation

The ANOVA conducted on the MRI RF values indicated significant main effects for the group (GAD vs. controls: *F*(1,98) = 13.89, *p* < 0.001, *η*^2^ = 0.12), treatment (before and after MoBa: *F*(1,98) = 15.47, *p* < 0.001, *η*^2^ = 0.14), and brain region (amygdala, hippocampus, and neocortex: *F*(2,196) = 70.10, *p* < 0.001, *η*^2^ = 0.42). The two-way interactions from this ANOVA were also significant: group by treatment (*F*(1,98) = 9.53, *p* < 0.01, *η*^2^ = 0.09), group by brain region (*F*(2,196) = 14.01, *p* < 0.001, *η*^2^ = 0.13), and treatment by group region (*F*(2,196) = 17.05, *p* < 0.001, *η*^2^ = 0.15). Critically, the three-way interaction, including group, treatment, and brain region, also reached the level of statistical significance (*F*(2,196) = 12.58, *p* < 0.001, *η*^2^ = 0.11).

**Figure 1:**
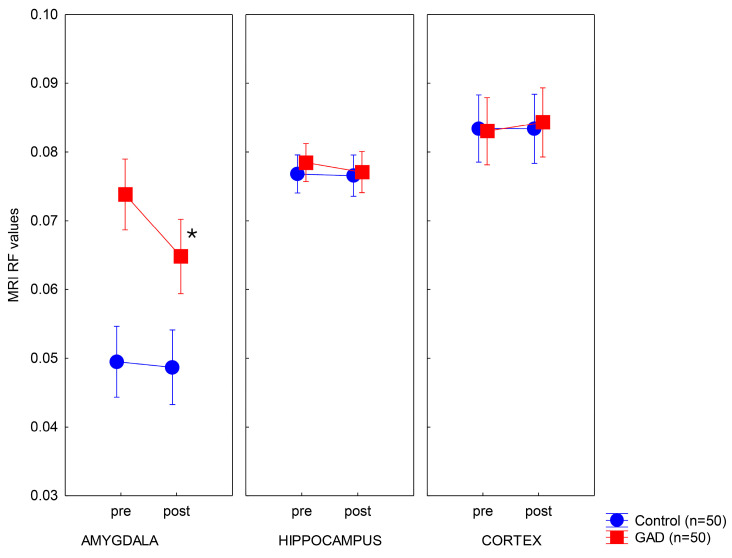
Restricted fraction (RF) values before (pre) and after (post) therapy. Data are mean. Error bars indicate 95% confidence intervals. * *p* < 0.001 (pre- vs. post-treatment).

Tukey’s HSD tests revealed that, before the treatment, amygdala RF values were elevated in the GAD group relative to the controls (*p* < 0.001). In contrast, there were no significant differences in the hippocampus and neocortex between patients with GAD and matched controls (*p*s > 0.5) (Figure 1). When the amygdala RF values measured before and after the treatment were compared, we observed a significant decrease in GAD, with markedly lower values after the treatment (*p* < 0.001). However, even after the treatment, patients with GAD still had significantly higher RF levels in the amygdala compared to the matched controls (*p* < 0.001), putatively indicating subtle signs of neuroinflammation (Figure 1). In the healthy control group, we observed remarkable stability and replicability of RF values across two measurements in all brain regions (*p*s > 0.8, Figure 1). 

### 3.3. Correlations between Changes in Clinical Symptoms and Inflammatory Markers

**Figure 2:**
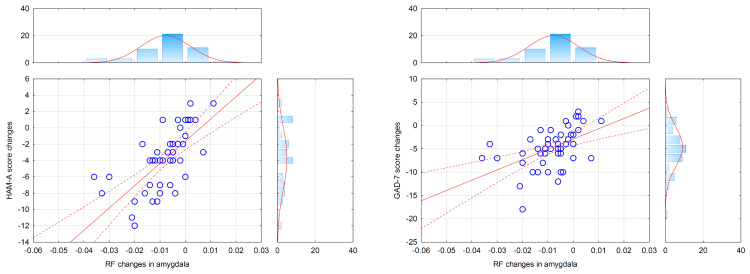
Correlations between changes in clinical scales of anxiety and restricted fraction (RF) values (after therapy minus before therapy). HAM-A, Hamilton Anxiety Rating Scale; GAD-7, General Anxiety Disorder-7; HAM-A—RF change correlation: *r* = 0.68; GAD-7—RF change correlation: *r* = 0.50, *p*s < 0.001. The blue circles represent individual cases, the red line indicates the correlation, and the red dotted line indicates 95% confidence intervals.

We calculated the correlations between HAM-A, GAD-7, and BDI-II scores changes during MoBa (post-treatment minus pre-treatment) and changes in RF values calculated with the same scheme (post-treatment minus pre-treatment). All correlation coefficients were corrected for age, education, and BMI. We found that changes in HAM-A and GAD-7 scores were significantly correlated with changes in RF values exclusively in the amygdala (HAM-A: *r* = 0.68, *p* < 0.001; GAD-7: *r* = 0.50, *p* < 0.001) (Figure 2). We found no significant relationships between BDI-II and RF values (*r* = −0.07, *p* > 0.2). 

### 3.4. Effects of Sex

We performed the above-described analyses with sex as a covariate and separately compared male and female participants. There was no significant evidence that male and female participants differed in any measures or that the results were impacted by sex effects (*p*s > 0.2). 

## 4. Discussion

The present study investigated the effects of MoBa on anxiety symptoms and neuroinflammatory markers in the hippocampus, amygdala, and neocortex in patients with GAD. The findings revealed significant reductions in both anxiety symptoms and neuroinflammatory markers in the amygdala following a 12-week course of MoBa. In addition, more pronounced anxiety reduction was linked to improved MRI neuroinflammatory markers. The observed reduction in anxiety symptoms is consistent with prior research demonstrating the efficacy of various forms of psychotherapy, particularly CBT, in treating GAD [8]. Intriguingly, we detected a more pronounced decrease in GAD-7 scores compared to HAM-A scores following MoBa treatment. This difference can be attributed to the GAD-7 scale’s specific focus on the core cognitive and affective symptoms of GAD, its sensitivity to changes in these symptoms, the self-report nature of the measure reflecting the patient’s perceived improvements, and the immediate impact of psychotherapy on cognitive symptoms. In contrast, the broader symptom range of the HAM-A, including somatic symptoms that may not improve as quickly, might result in smaller observable decreases in scores.

The prominent role of MoBa is especially relevant because the effectiveness of psychotherapy for adult depression and anxiety has been a topic of considerable debate, particularly following Eysenck’s claims in the mid-20th century that psychotherapy was ineffective [78]. Cuijpers et al. (2019) [79] conducted a comprehensive meta-analysis, re-evaluating the efficacy of psychotherapy by analyzing an extensive database of randomized trials from 1966 to 2017. They found a moderate effect size of 0.70 when psychotherapy was compared with control groups. However, after adjusting for publication bias and risk of bias, the effect sizes were reduced to small, suggesting that the real effectiveness of psychotherapy might be less than previously believed. In contrast, Munder et al. (2018) [80] reanalyzed Cuijpers et al.’s data [79], correcting for outliers and biases, and reaffirmed the effectiveness of psychotherapy, with a standardized mean difference of 0.70 compared to waiting list controls. This re-analysis provides evidence against Eysenck’s original assertion [78], establishing that psychotherapy remains a beneficial practice for treating adult mood and anxiety disorders, albeit with nuanced effectiveness when biases are fully considered. 

Also, the question remains of whether the waiting list and the poorly defined care-as-usual are appropriate control conditions in investigating psychotherapy’s effectiveness. The situation is especially important for anxiety disorders, including GAD, where the available evidence of psychotherapy effectiveness is less sound than for major depressive disorder [8]. In the present study, MoBa’s integrative feature of evidence-based psychotherapeutic techniques, such as CBT and mindfulness-based stress reduction, likely contributed to the marked improvements that we observed in our patients. This supports previous findings that structured and individually tailored therapeutic approaches can effectively alleviate anxiety symptoms [43]. 

To establish this individualized treatment approach, we used the RDoC framework, which offers a comprehensive approach to understanding GAD by focusing on core psychological processes across multiple levels of analysis [71]. It highlights the role of exaggerated threat responses mediated by neural circuits involving the frontal cortex, amygdala, hippocampus, and midbrain, influenced by genetic variations in the serotonin system and other neurotransmitters [81]. The framework also incorporates physiological dimensions of anxiety, revealing distinct patterns of defensive reactivity across anxiety disorders [82]. By integrating temporal dynamics and complex systems, RDoC enhances understanding of the maintenance and progression of anxiety [83]. It emphasizes combining observable behavior, neurobiological measures, and environmental influences to better understand the etiology of anxiety disorders, and examines avoidance and approach motivation through frontal EEG activity to gain insights into specific symptom clusters [84,85]. This integrative approach promises more precise diagnostics and personalized interventions based on individual neurobiological and behavioral profiles.

The reduction in neuroinflammatory markers in the amygdala aligns with emerging evidence suggesting that psychological interventions can influence biological processes, including inflammation [50,51,53]. A recent network meta-analysis evaluated the effects of various psychological interventions, such as cognitive therapy, mindfulness, and lifestyle changes, on adult immune system biomarkers [49]. Analyzing data from 104 randomized controlled trials involving 7820 participants, the study found that mindfulness-based interventions significantly reduced pro-inflammatory cytokines and increased anti-inflammatory cytokines. Cognitive therapy also showed a positive impact by reducing pro-inflammatory markers and increasing white blood cell count. The findings suggest that psychological interventions might improve immune function, particularly in individuals with inflammation-related conditions. Despite promising results, the study notes significant heterogeneity and calls for further research to strengthen the evidence [49].

Our findings contribute to the literature by demonstrating that MoBa, a multifaceted psychotherapeutic approach, can also reduce neuroinflammation in key brain regions associated with emotion regulation and anxiety. The strength of these findings is that it is the first demonstration of treatment-related reduction in inflammation in GAD at the level of anxiety-related brain structures. 

The significant correlation between the reduction in anxiety symptoms and changes in neuroinflammatory markers is noteworthy and warrants further consideration. Previous studies have documented associations between inflammation and psychiatric symptoms, and elevated levels of IL-6 and CRP have been linked to greater symptom severity in various anxiety disorders [13,17]. However, most of these studies focused on peripheral markers from blood samples, with a few exceptions [86]. Our findings raise the possibility that MoBa can reduce both anxiety symptoms and neuroinflammation, and these effects may occur through interdependent mechanisms. However, the complexity of GAD pathophysiology should not be neglected. GAD is a multifaceted disorder with a complex etiology involving genetic, environmental, psychological, and biological factors [3]. Neuroinflammation is only one of many potential contributors to the disorder. Therefore, MoBa’s efficacy in reducing anxiety symptoms may be mediated through pathways other than inflammation, such as changes in cognitive processes, stress reactivity, or neural connectivity.

Some limitations should be considered when interpreting the main findings of the study. First, while the sample size of 50 participants with GAD was adequate for detecting overall treatment effects, it may have been insufficient to detect more subtle relationships between changes in anxiety symptoms and neuroinflammatory markers. The sample was slightly underpowered (required sample: 63 participants in each group). More extensive studies are necessary to validate these findings and to explore potential moderating factors. Second, the study did not include a clinical control group receiving an alternative treatment or placebo (e.g., a supportive therapy or waiting list). Consequently, we cannot definitively attribute the observed changes to MoBa without ruling out potential placebo effects or natural symptom fluctuations over time. Future studies should include control conditions to strengthen causal inferences. Third, the follow-up period of 12 weeks might not be long enough to fully capture the long-term effects of MoBa on both anxiety symptoms and neuroinflammation. Extended follow-up periods are necessary to determine the durability of treatment effects and the potential delayed relationship between symptoms and inflammation. Finally, the study relied on a novel MRI to measure neuroinflammation [36,58,61]. While this method is robust, combining it with other biomarkers, such as blood cytokine levels or cerebrospinal fluid analyses, could provide a more comprehensive picture of the inflammatory processes involved.

## 5. Conclusions

This study demonstrated that algorithm-based modular psychotherapy effectively reduces both anxiety symptoms and neuroinflammatory markers in the amygdala in patients with GAD. The lessening of inflammation parallelled the improvement in symptoms. While these findings contribute to our understanding of the interplay between psychological and biological processes in GAD, they also highlight the complexity of the disorder and the need for further research. Future studies will elucidate the specific mechanisms of action, explore individual differences in treatment response, and assess the long-term efficacy of MoBa. By addressing these questions, we can move closer to developing more effective, personalized treatments for anxiety disorders, ultimately improving the quality of life for those affected.

## Figures and Tables

**Table 1:** Demographic characteristics and clinical scales.

	GAD (*n* = 50)		Healthy Controls (*n* = 50)	
Age (years)	39.9 (11.6)		39.6 (12.4)	
Education (years)	11.5 (3.5)		12.1 (3.2)	
Sex (male/female)	21/29		21/29	
Smoking (smokers/non-smokers)	17/33		17/33	
Alcohol consumption (units/week)	8.3 (5.8)		9.4 (5.4)	
Body mass index (BMI)	28.5 (9.1)		26.8 (8.8)	
Before and after MoBa	Before	After	Before	After
BDI-II	10.4 (6.0)	8.0 (4.7) *	-	-
HAM-A	23.2 (4.2)	19.1 (5.5) *	-	-
GAD-7	14.5 (3.9)	9.7 (5.0) *	-	-

Data are mean (standard deviation) except for sex distribution and smoking. BDI–II, Beck Depression Inventory-II; HAM-A, Hamilton Anxiety Rating Scale; GAD-7, General Anxiety Disorder-7; * Significantly lower values after therapy relative to the baseline (before therapy) (*p*s < 0.05).

## Data Availability

The raw data are available from the corresponding author upon request.
